# Malnutrition Risk among Older Mexican Adults in the Mexican Health and Aging Study

**DOI:** 10.3390/nu13051615

**Published:** 2021-05-12

**Authors:** Jaqueline C. Avila, Rafael Samper-Ternent, Rebeca Wong

**Affiliations:** 1 Center for Alcohol and Addiction Studies, School of Public Health, Brown University, Providence, RI 02903, USA; 2 Sealy Center on Aging, University of Texas Medical Branch, Galveston, TX 77555, USA; rasamper@utmb.edu (R.S.-T.); rewong@utmb.edu (R.W.); 3 Department of Internal Medicine, Division of Geriatrics, University of Texas Medical Branch, Galveston, TX 77555, USA; 4 Department of Preventive Medicine and Population Health, University of Texas Medical Branch, Galveston, TX 77555, USA

**Keywords:** malnutrition, older adults, Mexico, MNA, MHAS

## Abstract

Few studies assess the malnutrition risk of older Mexican adults because most studies do not assess nutritional status. This study proposes a modified version of the Mini Nutritional Assessment (MNA) to assess the risk of malnutrition among older Mexicans adults in the Mexican Health and Aging Study (MHAS). Data comes from the 2012, 2015, and 2018 waves of the MHAS, a nationally representative study of Mexicans aged 50 and older. The sample included 13,338 participants and a subsample of 1911 with biomarker values. ROC analysis was used to calculate the cut point for malnutrition risk. This cut point was compared to the definition of malnutrition from the ESPEN criteria, BMI, low hemoglobin, or low cholesterol. Logistic regression was used to assess predictors of malnutrition risk. A score of 10 was the optimal cut point for malnutrition risk in the modified MNA. This cut point had high concordance to identify malnutrition risk compared to the ESPEN criteria (97.7%) and had moderate concordance compared to BMI only (78.6%), and the biomarkers of low hemoglobin (56.1%) and low cholesterol (54.1%). Women, those older than 70, those with Seguro Popular health insurance, and those with fair/poor health were more likely to be malnourished. The modified MNA is an important tool to assess malnutrition risk in future studies using MHAS data.

## 1. Introduction

Malnutrition has been identified as a risk factor for adverse events among older adults. Further, malnutrition among older adults may present before a specific health condition [1], and having malnutrition is associated with poorer health outcomes, longer hospital stays, disability, and mortality [2,3,4]. Globally, there is a wide range of malnutrition prevalence among community older adults 60 years and older, ranging between 1.3% and 47.8% [5], with the greatest prevalence of malnutrition seen in low and middle-income countries [5]. A study among communities of older adults in 12 countries showed that the prevalence of malnutrition was 5.8% [6].

Low socioeconomic status [7,8] and poor health [9] have been reported as risk factors for malnutrition among older adults. Older adults in Mexico have aged in a health and socioeconomic context where these risk factors are highly prevalent, increasing the risk for malnutrition. First, a significant percentage of older adults in Mexico have low socioeconomic status. In 2012, nearly 10% of the population 50 and older reported 0 years of education and 17% of men and 14% of women did not have health insurance [10]. In addition, older adults in Mexico also have high prevalence of chronic diseases and disability that may add to their risk of malnutrition [10]. In 2012, more than half of the population 50 and older reported fair or poor health status and the prevalence of diabetes was near 20%. Further, 6.4% of men and 9.9% of women reported at least one limitation on Activities of Daily Living (ADL) [10]. Older adults in Mexico experienced the earlier stages of the epidemiologic transition with high prevalence of infectious diseases and childhood malnutrition, and they aged as the transition occurred. These adults, therefore, experience a double disease burden (infectious and chronic) that results in poor outcomes [11,12]. They experience high prevalence of chronic diseases, in addition to high disease-related disability and mortality, due to poor disease management and low access to care.

Despite the greater malnutrition risk of older Mexican adults [13], few studies have focused on the malnutrition risk of this population, and most studies assessing nutritional status focus on the high prevalence of overweight and obesity in this population [14,15]. Although the older Mexican population has high obesity rates, malnutrition and obesity can coexist [16,17].

One of the barriers for the study of malnutrition among older adults in Mexico is that the longest running nationally representative survey of the population 50 and older in Mexico, the Mexican Health and Aging Study (MHAS), does not assess nutritional status directly. The MHAS is a comprehensive population-based study of aging and health in Mexico, thus limiting the opportunity for a full nutritional assessment. Although the MHAS does not apply a full nutritional assessment, it includes several measures that, when used together, result in a modified version of the Mini Nutritional Assessment (MNA). The MNA is a widely used tool to screen the nutritional status of community-dwelling older adults, and it provides a validated and detailed assessment that goes beyond considering BMI and weight loss only [18,19]. Thus, the first objective of this paper is to use MHAS data to propose a modified MNA to identify older Mexican adults at risk for malnutrition. The modified MNA includes determination of a cut point to classify older Mexican adults in the MHAS as at risk for malnutrition. In addition, the modified MNA will be compared with other measures of nutritional status such as objective biomarkers, body mass index (BMI), and the European Society of Parenteral and Enteral Nutrition and Metabolism (ESPEN) criteria. The second objective is to use the modified MNA to assess the main predictors of malnutrition risk among older Mexican adults.

## 2. Methods

### 2.1. Data

We used the 2012 wave of the MHAS to conduct this study to provide a nationally representative cross-section of the population aged 50 and older in Mexico. We also used the 2015 and 2018 waves of the MHAS to provide data on mortality for those in the 2012 wave. In addition, we used a subsample of the 2012 cohort that provided a blood sample to measure biomarkers and measured height and weight. The final sample included 13,338 individuals aged 50 and older on whom we had all the information needed to assess nutritional status. The sample size of the subsample for the biomarker analysis was 1911 individuals.

#### 2.1.1. Development of the Modified Mini Nutritional Assessment

Table 1 shows the modifications made to the original short-form MNA to allow it to be used with the MHAS. The original MNA includes measures related to changes in food intake, weight loss, mobility, stress and acute disease, neuropsychological disorders, and BMI over the past three months. In the original MNA, each measure receives a score and these are summed to a total score ranging from 0 to 14 [19,20]. Similar measures capturing the same domains were assessed in the MHAS survey. These measures were scored in the same way as the original MNA and were summed into a total score ranging from 0 to 14.

One main difference between the MNA and the modified MNA is the time reference: in the MHAS, respondents are asked similar questions but using the previous two years as the point of reference. In addition, there were differences on specific items. For example, the weight loss item in the modified MNA was assessed as change in 5 kg or more instead of 3 kg or more. The MHAS only assessed changes of 5 kg or more and did not assess if there was any change between 1 and 5 kg. Thus, no one received a score of “2” in the modified MNA. The stress and acute disease item was assessed with the closest measure of psychological distress in the MHAS (report of a major event such as accident, crime, or natural disaster), and the closest measure of acute disease (hospitalization). Due to differences in the time span, as well as other differences on specific items asked between the original MNA and the MHAS modification, we did not consider the cut points of malnutrition, at risk for malnutrition, and normal nutrition from the original MNA.

We used receiver operating characteristic (ROC) analysis to calculate the optimal cut point for malnutrition risk. In order to create the ROC curve, we examined the effectiveness of the modified MNA in 2012 to predict mortality by 2018. We calculated the ROC curve based on mortality because this is an outcome highly associated with malnutrition [4] and serves as a close proxy when malnutrition is not assessed. Evidence from a systematic review showed that the original short-form MNA significantly predicted mortality over time among community older adults [21]. The Liu index was used to determine the best cut point based on the maximized sensitivity and specificity. We generated 1000 bootstrap samples to internally validate the cut point of the modified MNA and calculated the area under the curve (AUC), sensitivity, specificity, positive predictive value and negative predictive value at the optimal cut point.

We also conducted logistic regression models to assess the odds of dying with a 1-unit increase in the modified MNA score, as well as the odds of dying for those identified as at risk for malnutrition compared to those with normal nutritional status, based on the cut point determined as described above.

Sensitivity Analysis: Comparison to other nutritional screenings.

After the optimal cut point was defined, we conducted sensitivity analysis to compare the concordance of the modified MNA cut point to other validated measures used to assess nutritional status: the ESPEN criteria, BMI, and biomarkers. This sensitivity analysis was restricted to the subsample with biomarker information. We compared how many of those classified at risk for malnutrition, based on the modified MNA, were also classified as with malnutrition based on these measures.

- ESPEN criteria: The ESPEN criteria categorizes older adults at risk for malnutrition if either of these alternatives is met [22]:
  (1) BMI 18.5 kg/m^2^
  (2) Unintentional weight loss greater than 10% over an indefinite time, or 5% in the past 3 months *in addition* to either of these conditions:
    (2a) BMI 20 if younger than 70, or BMI 22 if 70 or older
    (2b) Fat Free Mass Index (FFMI) 15 for women and 17 for men

We used both alternatives to classify older adults using the MHAS data. The BMI values used for the first alternative were obtained using measured height and weight and imputed for missing values by the MHAS research team. In the second alternative, unintentional weight loss was defined as answering yes to the question “weight loss greater than 5 kg in the previous two years” to meet the MNA criteria for weight loss greater than 10% over an indefinite time. We considered that 5 kg or more is more than 10% of weight, given that the mean weight in the MHAS population is 70 kg (SD: 13.7). The second alternative was classified based on the 2a alternative of BMI differences, considering age. This is because FFMI (alternative 2b) is not measured in the MHAS. Individuals who met alternatives 1 or 2 were defined as at risk for malnutrition.

- Biomarkers: We used low cholesterol or low hemoglobin as proxies for malnutrition as previous studies have indicated [23]. Participants with cholesterol ≤160 mg/dL, or hemoglobin ≤12 for women and ≤13 for men were defined as at risk for malnutrition.
- BMI only: We used underweight, defined as BMI 18.5 kg/m^2^, as a proxy for malnutrition.

#### 2.1.2. Analysis of Predictors of Malnutrition among Older Mexican Adults

We used a logistic regression model to assess the predictors of malnutrition risk using the modified MNA. We included the following demographic and health covariates associated with malnutrition: sex, age (50–59; 60–69; 70+ years), education (0; 1–6; 7+ years), health insurance status (None; IMSS/ISSTE/Other; Seguro Popular), locality size (less urban if population 100,000; more urban if population ≥ 100,000), and self-reported health (Excellent/Very good/Good; Fair; Poor).

## 3. Results

### 3.1. Accuracy of the Modified MNA

The sample characteristics are described in Supplementary Table S1. Overall, the majority of older Mexican adults were female, ages 50–59, and had 1–6 years of education. Near 15% of the sample did not have health insurance, and 12% reported poor health status.

The results from the ROC analysis showed that the best cut point for the modified MNA score was 10.5, which was rounded down to 10. Those with an MNA score of 0–10 were defined as at risk for malnutrition, and those with scores 11–14 were defined as having normal nutritional status. A total of 40.4% of the sample was classified as at risk for malnutrition. This cut point (0–10) was able to predict mortality by 2018 with moderate to high accuracy. The AUC for having a score of 10 or lower was 0.61, the sensitivity was 60.5%, the specificity was 62.5%, the positive predictive value was 18.8%, and the negative predictive value was 91.7%.

A 1-point increase in the modified MNA scale was associated with a 15% decrease in the 6-year odds of mortality (Odds Ratio (OR): 0.85, 95% Confidence Interval (CI): 0.83; 0.87) (Table 2) and those classified as at risk for malnutrition based on the proposed cut point were 79% more likely to die by 2018 than those with normal nutritional status (OR: 1.79, 95% CI: 1.59; 2.01) (Table 2).

Further, when compared to other measures of nutritional status, the modified MNA cut point had moderate to high concordance. In the subsample with biomarker information, the sensitivity of the modified MNA cut point was 93.2% compared to the ESPEN criteria, 78.6% compared to BMI of underweight, 54.1% compared to the objective marker of low cholesterol, and 56.1% compared to low hemoglobin. The specificity of the cut point was also moderate, close to 60% for all of the measures (Table 3).

### 3.2. Predictors of Malnutrition Risk

Results based on the modified MNA (Table 4) showed that women were more likely to have malnutrition than men. Higher odds of malnutrition were associated with older age and with fewer years of educational achievement. Further, older adults with Seguro Popular as health insurance were 17% more likely to be at risk of malnutrition compared to those with other types of health insurance (IMSS/ISSSTE/Other). Self-reported health was strongly associated with malnutrition in the modified MNA, where individuals who reported fair health were 2.4 times more likely to be at risk for malnutrition (OR: 2.39, 95% CI: 2.19; 2.60), and those who reported poor health were 6.8 times more likely to be at risk for malnutrition compared to those with either excellent, very good, or good health (OR: 6.84, 95% CI: 6.02; 7.78).

## 4. Discussion

The results from this study show that the modified MNA, using MHAS data, can be used as an important source of information regarding the nutritional status of older Mexican adults. Our evidence shows that the modified MNA was significantly associated with greater odds of 6-year mortality as a total continuous score, as well as based on the proposed cut point for “at risk for malnutrition”.

The proposed cut point had high sensitivity and moderate specificity to predict 6-year mortality. The modified MNA performed with high sensitivity compared to other measures of nutritional status, such as the ESPEN criteria and BMI of underweight only. More than 90% of those classified as at risk for malnutrition with the modified MNA were also classified as malnourished based on the ESPEN criteria. The sensitivity of the modified MNA cut point was lower for the BMI of underweight only, biomarkers of malnutrition (low cholesterol and low hemoglobin), but had moderate concordance with these (78.6%, 54.1% and 56.1%, respectively). The specificity of the modified MNA was also moderate/low compared to these measures.

Based on the modified MNA, we estimate that 40% the MHAS sample is at risk for malnutrition. Evidence from a study of Mexican adults 60 years and older from the U.S./Mexico border that applied the original MNA also showed half of the sample was at risk for malnutrition (50.2%) and that the prevalence of malnutrition was 8.6% for women and 6.3% for men [24]. Another study in Mexico City found that the prevalence of malnutrition risk was 31.8% among Mexican adults 60 years and older using the in-depth assessment of the MNA (not only the screening part used here) [25]. Evidence from a study combining data from community older adults in 12 countries showed that that 31.9% of older adults were at risk for malnutrition [6].

The results of this study show that the predictors of malnutrition risk, in older Mexican adults, include both socioeconomic and health factors. The main predictors of malnutrition risk were having Seguro Popular insurance, having lower education, and reporting fair or poor health. Previous work in Mexico also highlighted that, in addition to similar health risk factors, socioeconomic factors, such as not having a pension, reporting lack of money to live on [25], and illiteracy [24] were also risk factors for malnutrition in this population. Studies in Spain and Italy also showed that low educational level and other measures of low SES were also associated with greater malnutrition risk among older adults, but this effect was largely explained after adjusting for sex and age [7,8]. Studies in the U.S. showed that socioeconomic factors do not appear to be main risk factors for malnutrition among older adults. The main risk factors in the U.S. were declining health status, poor cognition, and eating problems, such as difficulty swallowing [9].

Measuring nutritional status can be demanding in population-based surveys. One of the strengths of this study is that we used data from a nationally representative study of older Mexican adults that does not directly evaluate nutritional status, and we modified the MNA for potential incorporation into research of nutritional status among older adults in Mexico. The results from this study also help identify target populations in Mexico for interventions to potentially decrease the risk of malnutrition.

## 5. Limitations

Our approach has limitations, such as the inability to assess further nutritional information, such as short-term changes, and whether weight loss was intentional or unintentional. Further, the MNA cut point proposed in this study has low specificity and may overestimate false positives. This low specificity is seen because mortality is only a proxy for malnutrition. This measure would likely be higher if nutritional status was measured with the original MNA and compared to this modified version. Because of this limitation, the proposed cut point should not be used to assess the prevalence of malnutrition in the population but only as a marker for malnutrition risk. In addition, we recommend that, if the MHAS user wants to study malnutrition risk with the cut point of 10, they should acknowledge the limitation of the sensitivity and specificity of the proposed cut point. We recommend that one way to avoid this limitation is to treat the modified MNA as a continuous score.

## 6. Conclusions

In summary, our study showed that approximately 40% of Mexican adults aged 50 and older are at risk for malnutrition, according to the modified MNA. Further, the main risk factors for malnutrition in this population include dimensions of both socioeconomic and health status. The modified MNA proposed in this study can be used to capture a comprehensive picture of malnutrition risk among older adults in Mexico and can inform future work with the MHAS aiming to incorporate nutritional status, thus contributing with a framework that is replicable by other investigators.

## Figures and Tables

**Table 1:** Measures for the Mini Nutritional Assessment Adaptation using MHAS 2012.

Original Mini Nutritional Assessment	Modified MNA Using MHAS
**Has food intake declined over the past 3 months due to loss of appetite, digestive problems, chewing or swallowing difficulties?**	**In the last two years, respondent has eaten less due to loss of appetite, digestive problems, and difficulties chewing or swallowing**
0 = Severe decrease in food intake	0 = Most of the time
1 = Moderate decrease in food intake	1 = Sometimes
2 = No decrease in food intake	2 = Hardly ever
**Weight loss during the last 3 months**	**Respondent has experienced weight loss in the past 2 years**
0 = Weight loss greater than 3 kg	0 = Decreased 5kg or more
1 = Does not know	1 = Does not know
2 = Weight loss between 1 and 3 kg	2 = N/A
3 = No weight loss	3 = Remained the same or increased 5kg or more
**Mobility**	**Respondent’s mobility**
0 = Bed or chair bound	0 = Has difficulty getting in and out of bed AND has difficulty walking one block or several blocks
1 = Able to get out of bed/chair but does not go out	1 = Does not have difficulty getting in and out of bed AND has difficulty walking one block or several blocks OR has problems getting in and out of bed AND does not have difficulty walking one block or several blocks
2 = Goes out	2 = Does not have difficulty walking one block or several blocks AND does not have difficulty getting in and out of bed
**Has suffered psychological stress or acute disease in the past 3 months?**	**Respondent has suffered a major event with psychological trauma from accident, crime or natural disaster or has been hospitalized in the past 2 years**
0 = Yes	0 = Has suffered a natural disaster that damaged home OR suffered accident, crime, or any similar event OR has been hospitalized in the last 2 years
2 = No	2 = Has not suffered a natural disaster that damaged home AND has not suffered accident, crime, or any similar event AND has not been hospitalized in the last 2 years
**Neuropsychological problems**	**Respondent’s current memory status and depressive symptoms**
0 = Severe dementia or depression	0 = Has poor memory AND depressive symptoms
1 = Mild dementia	1 = Has a fair memory AND does not have depressive symptoms
2 = No psychological problems	2 = Has a good memory AND does not have depressive symptoms
**Body Mass Index (BMI)**	**BMI (self-report of weight and height)**
0 = BMI less than 19	0 = BMI less than 19
1 = BMI 19 to less than 21	1 = BMI 19 to less than 21
2 = BMI 21 to less than 23	2 = BMI 21 to less than 23
3 = BMI 23 or greater	3 = BMI 23 or greater
Screening Score	
12–14 Points: Normal nutritional status	11–14: Normal nutritional status
8–11 point: At risk of malnutrition	
0–7 points: Malnourished	0–10: At risk for malnutrition

**Table 2:** Logistic regression models to test the association between the modified MNA as a continuous score, with the proposed cut-point, and 6-year mortality in 2015. 2012 MHAS Sample (*n* = 13,338) ^a^.

	Continuous MNA Score		At Risk for Malnutrition (Proposed Cut-Point)	
	OR	95% CI	OR	95% CI
Modified MNA Score	0.85 ***	0.83; 0.87	NA	
Modified MNA Cut-off				
0–10 (At risk for malnutrition)	NA		1.79 ***	1.59; 2.01
11–14				Ref.

^a^ Results adjusted by sex, age, locality size, education, health insurance, and self-reported health status. *** *p*-value 0.001. NA: Not applicable.

**Table 3:** Concordance of malnutrition risk in the modified MNA compared to other markers of malnutrition in the MHAS 2012 biomarker subsample (*n* = 1911) ^a^.

Modified MNA	ESPEN Criteria		BMI Only		Low Cholesterol		Low Hemoglobin	
	Yes (%)	No (%)	Yes (%)	No (%)	Yes (%)	No (%)	Yes (%)	No (%)
At risk for malnutrition	93.2	38.3	78.6	39.3	54.1	38.0	56.1	38.7
Normal nutritional status	6.8	61.7	21.4	60.7	45.9	62.0	43.9	61.3

^a^ Results shown in column percentages.

**Table 4:** Predictors of Malnutrition Risk in the MHAS 2012.

	At Risk for Malnutrition (Yes vs. No)	
	OR	95% CI
Female (Ref: Male)	1.41 ***	1.30; 1.52
Age (Ref: 50–59)		
60–69	1.23 ***	1.12; 1.35
70+	1.92 ***	1.74; 2.12
Education years (Ref: 0 years)		
1–6	0.86 ***	0.77; 0.95
7+	0.65 ***	0.58; 0.74
Insurance Status (Ref: IMSS, ISSSTE, Other)		
Uninsured	0.92	0.81; 1.04
Seguro Popular	1.17 ***	1.06; 1.28
More Urban (Ref: Less Urban)	1.11 **	1.02; 1.21
Self-reported health (Ref: Excellent, very good or good)		
Fair	2.39 ***	2.19; 2.60
Poor	6.84 ***	6.02; 7.78

** *p*-value 0.01; *** *p*-value 0.001.

## Data Availability

Data from the MHAS is publicly available at mhasweb.org, accessed data 11 May 2021.

## Supplementary Materials

The following are available online at https://www.mdpi.com/article/10.3390/nu13051615/s1, Table S1: Weighted Sample Characteristics of older Mexican adults in the 2012 MHAS.
