# Supplementary material for: Malnutrition Risk among Older Mexican Adults in the Mexican Health and Aging Study
Source: Nutrients. 2021 May 12;13(5):1615. doi: 10.3390/nu13051615 (PMC8151238; doi:10.3390/nu13051615)
Supplement: Supplementary file 1 [file nutrients-13-01615-s001.zip › nutrients-1169427-supplementary.pdf]

Table S1. Weighted Sample Characteristics of older Mexican adults in the 2012 MHAS.

Characteristics	N	%
**Sex**		
Male	5703	46.0
Female	7635	54.0
**Age**		
50-59	4391	46.1
60-69	5020	31.8
70+	3927	22.1
**Education years**		
0	2276	16.4
1-6	6929	50.9
7+	4073	32.7
**Insurance Status**		
IMSS, ISSSTE, Other	1622	53.7
Seguro Popular	3800	31.4
Uninsured	1622	14.9
**Locality Size**		
More Urban	7876	50.3
Less Urban	5382	49.7
**Self-reported Health**		
Excellent, Very good, or Good	4786	37.1
Fair	6813	50.3
Poor	1736	12.3
